# Downregulated Serum Exosomal miR-451a Expression Correlates With Renal Damage and Its Intercellular Communication Role in Systemic Lupus Erythematosus

**DOI:** 10.3389/fimmu.2021.630112

**Published:** 2021-02-12

**Authors:** Lina Tan, Ming Zhao, Haijing Wu, Yuezhong Zhang, Xiaoliang Tong, Lihua Gao, Lu Zhou, Qianjin Lu, Jinrong Zeng

**Affiliations:** ^1^Department of Dermatology, Third Xiangya Hospital, Central South University, Changsha, China; ^2^Department of Dermatology, Second Xiangya Hospital, Central South University, Changsha, China; ^3^Xiangya School of Medicine, Central South University, Changsha, China

**Keywords:** exosome, microRNA-451a, serum, renal damage, SLE

## Abstract

Systemic lupus erythematosus (SLE) is a multi-system autoimmune disease characterized by continuous inflammation and the production of autoantibodies. Exosomes, acting as a critical tool for communication between cells, are involved in the pathogenesis of SLE, particularly in inflammation and immune imbalance. In this study, we aimed to extract and confirm the pro-inflammatory effect of serum exosomes in SLE. Then, we attempted to find differentially expressed exosomal microRNAs in the serum of healthy subjects and SLE patients *via* miRNA microarray analysis and validated the target exosomal microRNA, exosomal miR-451a, which expression level decreased in serum of SLE patients by RT-qPCR. Furtherly, we analyzed the correlation between exosomal miR-451a and disease activity, kidney damage and typing, and traditional medicine therapy. Finally, we investigated the intercellular communication role of exosomal miR-451a in SLE by co-culture assay *in vitro*. Taken together, our study demonstrated that downregulated serum exosomal miR-451a expression correlated with SLE disease activity and renal damage as well as its intercellular communication role in SLE which provided potential therapeutic strategies.

## Introduction

Systemic lupus erythematosus (SLE) is an autoimmune-induced diffuse connective tissue disorder associated with immune inflammation, which involves multiple systems and organs, and leads to significant morbidity and mortality (1, 2). Serological biomarkers are crucial for achieving timely diagnosis and precise evaluation of SLE (3). Recent studies have identified several new biomarkers that can be useful in clinical applications. For example, microRNAs (miRNAs or miRs), exosomes and exosomal miRNAs can all serve as biomarkers in autoimmune diseases (4). Published evidence indicates that miRNAs participate in different immune responses (5, 6), which makes them crucial candidates for use as diagnostic markers and treatment targets (7, 8). Increasing evidence suggests that the expression characteristics of serum miRNAs makes them possible biomarkers of autoimmune diseases, including SLE, and therefore they should be exploited for the betterment of humankind (9, 10).

Exosomes are membrane-bound microvesicles of 30–100 nm in diameter serving as small membranous transport vesicles, which are formed in the cytoplasm and released from the surface of almost all living cells (11, 12). Therefore, exosomes are found in numerous different body fluids, such as breast milk, serum, urine, and saliva (13). Exosomes consist of a lipid bilayer, containing miRNAs, mRNAs, and proteins. The lipid bilayer shields the genetic materials inside, thus protecting them from damage (14). In addition, previous evidence indicates that the majority of miRNAs in serum are deposited in exosomes (15). Therefore, owing to the aforementioned characteristics of exosomes, exosomal miRNAs in serum have been suggested as novel biomarkers that can be applied in the diagnosis and evaluation of disease outcomes for various conditions (4, 16–18). However, there have been relatively few studies on exosomal miRNAs in SLE. Solé et al. (19, 20) found that a multimarker of urine exosomes composed of miR-150, miR-21, and miR-29c provides a method for detecting early renal fibrosis and for predicting the progression of lupus nephritis (LN) disease. Compared with normal controls and non-LN patients with SLE, patients with active LN exhibited considerably higher exosomal miR-146a expression in urine (21). Lee et al. (22) found that circulating exosomes had immunological activity, and their levels reflected how active the disease is in patients with SLE. However, specific miRNAs in serum exosomes associated with renal damage have not been adequately investigated in SLE. Since the pathogenesis of abnormal immune function in SLE is extremely complex, there may be additional miRNAs involved in its immune pathogenesis. Moreover, the production, secretion, and transfer of exosomes take part in the intercellular communication system to induce immune dysregulation events. Exosomes serve as a messenger between maternal cells and recipient cells by transporting their cargo, including miRNAs (23). Approximately 10–15% of circulating miRNAs are protected from degradation through inclusion in exosomes (24).

The present study employed microarray analysis to assess the serum exosomal miRNA expression profiles of patients with active and inactive SLE vs. healthy controls. Based on our results, it was found that miR-451a was downregulated in serum exosomes of patients experiencing active SLE. Subsequently, the expression level of serum exosomal miR-451a in patients with SLE, and its association with renal damage and pathology type were further studied. Furthermore, the role of miR-451a transported by exosomes in lymphocyte communication was observed *in vitro*.

## Results

### Characteristics and Functions of Exosomes Isolated From Serum in SLE

**Figure 1:**
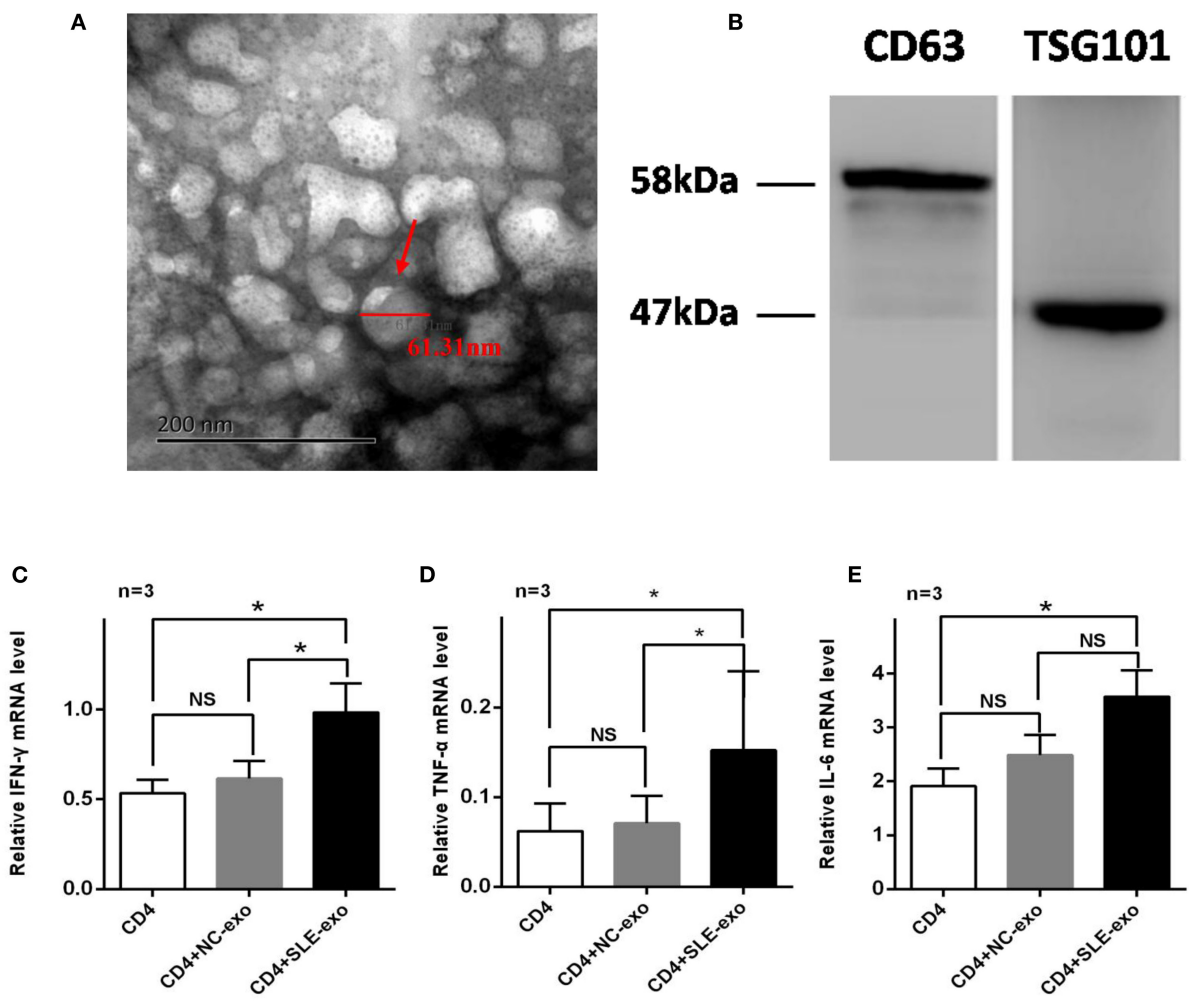
Validation and function of exosomes extracted from serum samples. **(A)** Transmission electron microscopy (*n* = 3) revealed the spherical characteristics and size (30–110 nm) of exosomes by ultracentrifugation pellet from serum. **(B)** Western blotting (*n* = 3) showed that the pellet after ultracentrifugation was strongly reactive for antibodies against the exosomal membrane markers CD63 and TSG101. Reverse transcription-quantitative PCR (*n* = 3) was employed to detect the mRNA expression levels of **(C)** TNF-α, **(D)** IFN-γ, and **(E)** IL-6 in CD4^+^ T cells co-cultured with exosomes of normal controls(NC) or patients with systemic lupus erythematosus(SLE) for 24 h, The mRNA expression levels of TNF-α, IFN-γ, and IL-6 were increased significantly after treated with SLE-exo while no significant changes in NC-exo group. RT-qPCR results were normalized to GAPDH. *P < 0.05, **P < 0.01, NS, no significance.

**Figure 2:**
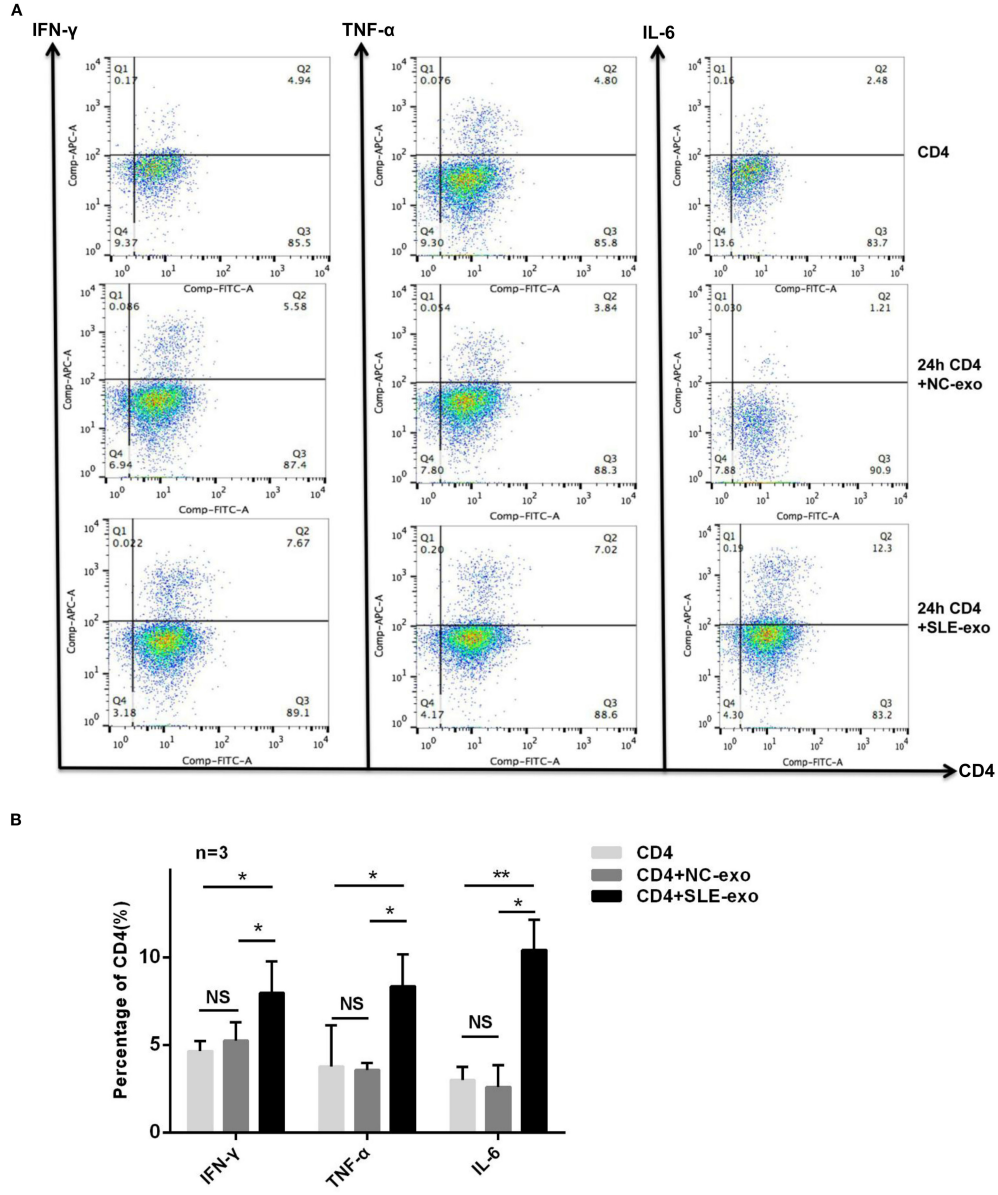
Further investigation of exosomes function by flow cytometry. **(A)** CD4 and anti-IFN-γ, TNF-α, or IL-6 antibody double labeling of cells was used to distinguish differential expression in CD4^+^ T cells before co-culture and after 24 h co-culture with exosomes of normal controls (NC) or patients with SLE *in vitro*. **(B)** Statistical analysis of the difference in expression of IFN-γ (*n* = 3), TNF-α (*n* = 3), and IL-6 (*n* = 3) by flow cytometry in CD4^+^ T cells and the results revealed the expression of IFN-γ, TNF-α, and IL-6 in CD4^+^ T cells co-cultured with exosomes of SLE was significantly higher than CD4^+^ T cells before co-culture or CD4^+^ T cells co-cultured with exosomes of NC, while the CD4^+^ T cells co-cultured with exosomes of SLE group and CD4^+^ T cells co-cultured with exosomes of NC group showed no differences. *P < 0.05, **P < 0.01, NS, no significance.

Western blotting and TEM were used to validate the effectiveness of the serum exosome extraction technique applied herein. The TEM results indicated that the exosomes extracted from the serum samples exhibited a spherical structure, with a size range of 30–110 nm (Figure 1A), a feature that was similar to those reported previously (12, 25). Through testing, it was further confirmed that these vesicles were exosomes. Western blot analysis was used to test specific exosomal markers (i.e., the tetraspanin molecules CD63 and TSG101) (Figure 1B). Moreover, it was investigated whether circulating active SLE exosomes could boost the SLE-specific effects of inflammatory response with an *in vivo* experiment. Thus, healthy CD4^+^ T cells treated with 10 μl exosomes purified from NCs (*n* = 9) or patients with active SLE (*n* = 9) were sorted. Compared with the NCs exosomes, the active SLE exosomes induced CD4^+^ T cells to produce significantly higher levels of IFN-γ, TNF-α, and IL-6, according to the results of RT-qPCR (Figures 1C–E) and flow cytometry (Figures 2A,B).

### Screening and Identification of Differentially Expressed Serum Exosomal miRNAs in Patients With SLE

**Figure 3:**
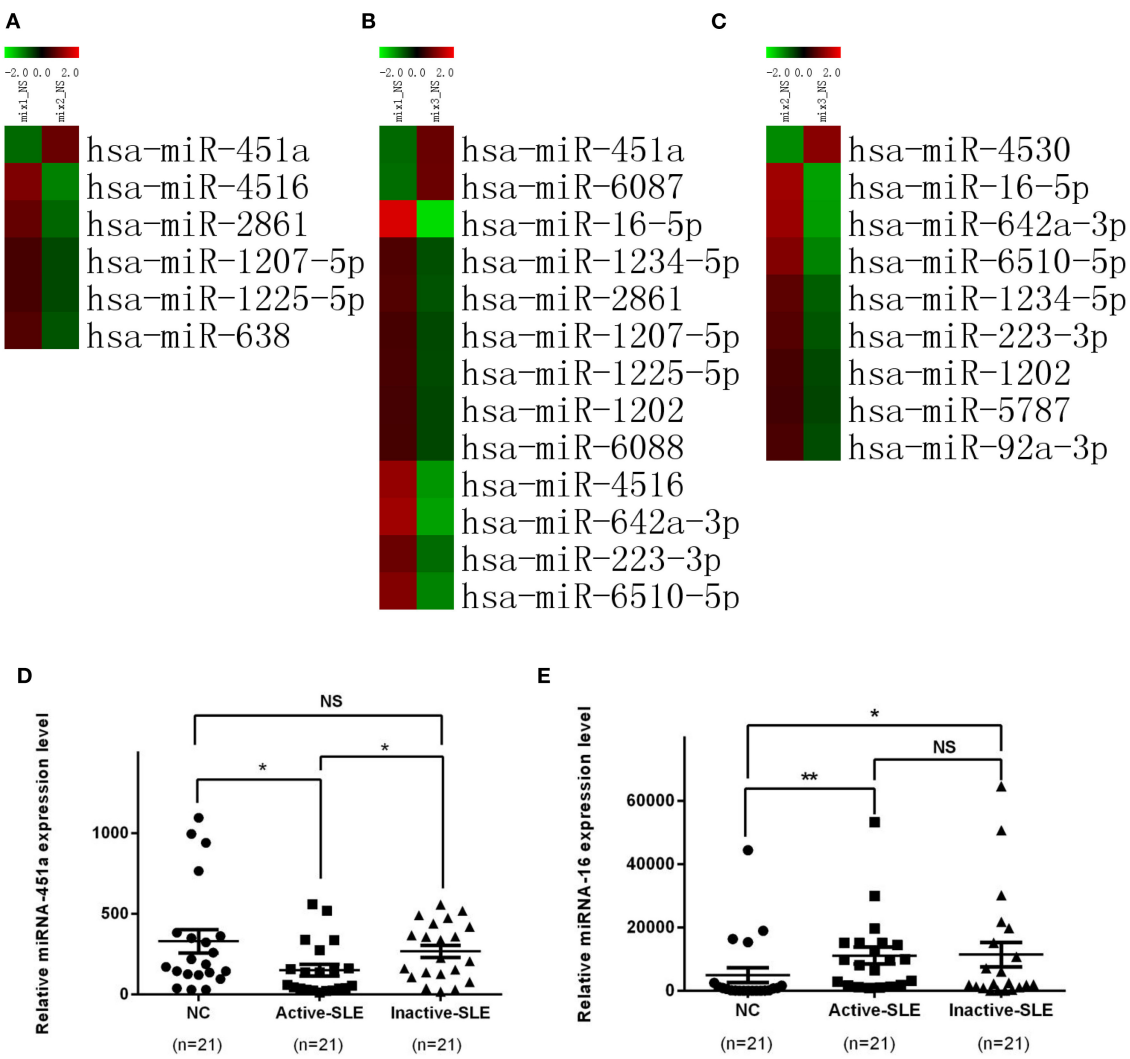
Significant differential expression of miRNAs in serum exosomes in patients with SLE and normal controls. **(A)** Heat map comparing active SLE and normal control groups. **(B)** Heat map comparing active and inactive SLE groups. **(C)** Heat map comparing inactive SLE and normal control groups. **(D)** The expression level of exosomal miR-451a in active SLE (*n* = 21) was lower than that in patients with inactive SLE (*n* = 21) and normal controls (*n* = 21), while the expression level of exosomal miR-451a in patients with inactive SLE and normal controls showed no differences. **(E)** The expression level of exosomal miR-16 in active SLE (*n* = 21) and inactive SLE (*n* = 21) was higher than that in normal controls (*n* = 21), but showed no significant difference between patients with active SLE and inactive SLE. RT-qPCR results were normalized to Cel-miR-39. *P < 0.05,**P < 0.01, NS, no significance.

Based on the above results, a miRNA microarray was performed to screen differentially expressed serum exosomal miRNAs in serum of healthy subjects vs. active or inactive SLE patients. A fold change >2 (upregulated) or <0.5 (downregulated) in the miRNA microarray data indicated a significant difference in miRNA expression. The raw data of miRNA microarray had been submitted to Gene Expression Omnibus (GEO accession: Agilent-070156). Significantly different expression profile of miRNAs in the serum exosomes of the three groups (active, inactive and healthy control samples) are shown in Figures 3A–C. Compared with the normal control group, 11 miRNAs, namely miR-1202, miR-1207-5p, miR-1225-5p, miR-1234-5p, miR-16-5p, miR-223-3p, miR-2861, miR-4516, miR-6088, miR-642a-3p, and miR-6510-5p, were significantly up-regulated in the serum exosomes from active SLE patients (*P* < 0.01) while miR-451a and miR-6087 were significantly down-regulated (*P* < 0.01). Compared with inactive SLE patients, 5 miRNAs including miR-1207-5p, miR-1225-5p, miR-2861, miR-4516, and miR-638 were significantly up-regulated (*P* < 0.01) in serum exosomes from active SLE patients, while miR-451a was significantly down-regulated (*P* < 0.01). Compared with the normal controls, the expression of miRNAs, including miR-1202, miR-1234-5p, miR-16-5p, miR-223-3p, miR-5787, miR-642A-3p, miR-6510-5p, and miR-92a-3p, were significantly up-regulated (*P* < 0.01) in inactive SLE patients and miR-4530 was significantly down-regulated (*P* < 0.01).

Since the expression of miR-451a and miR-16 showed great difference SLE patients and healthy controls, we selected them as the targets for further verification by RT-qPCR in samples from 21 patients with active SLE, 21 patients with inactive SLE and 21 normal control subjects (Figures 3D,E). However, only exosomal miR-451a was differentially expressed between active SLE patients and inactive SLE patients (*P* < 0.05). These results suggested that exosomal miR-451a might be correlated with SLE diseases activity.

### Serum Exosomal miR-451a Correlates With SLE Disease Activity and Renal Damage

**Figure 4:**
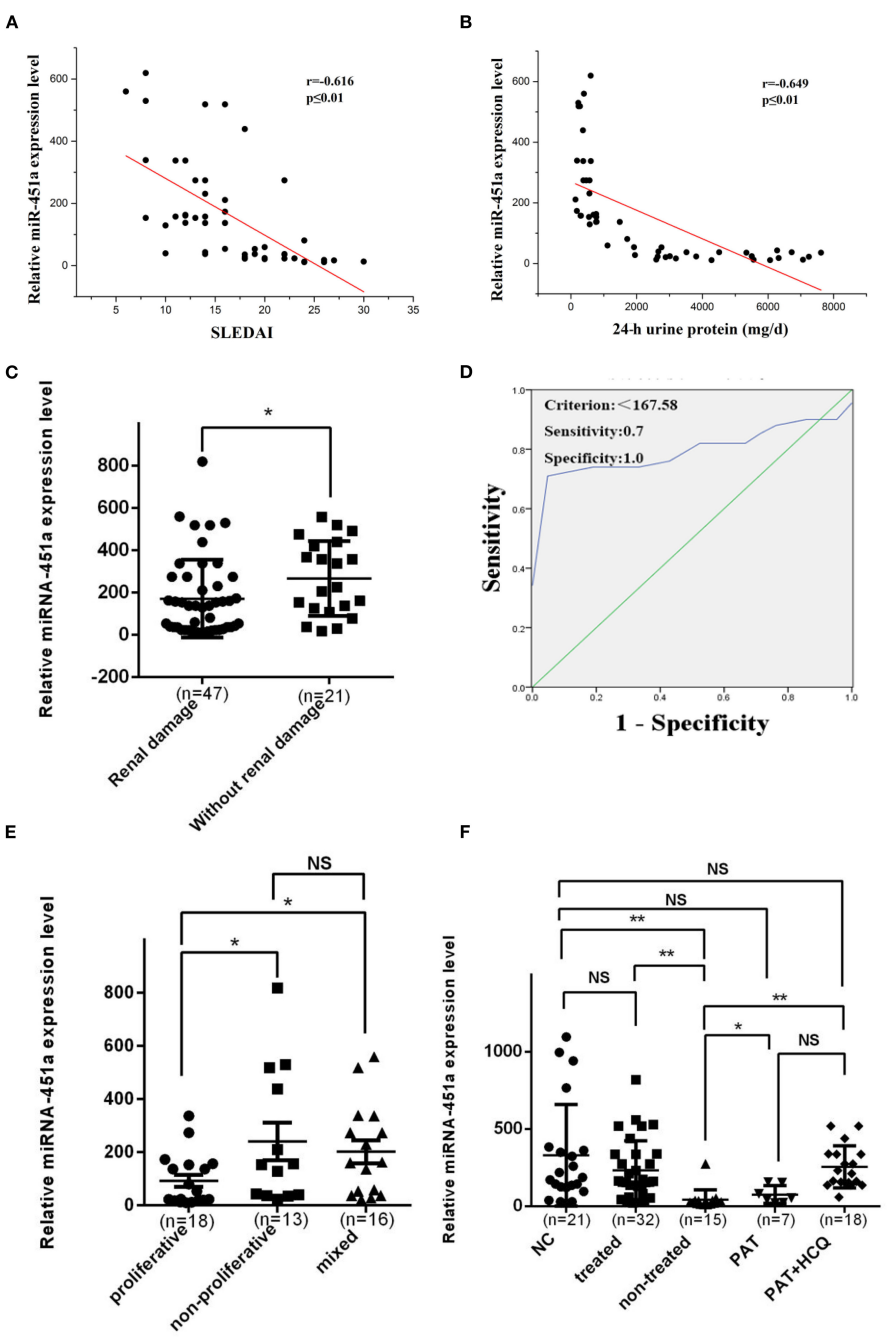
Serum exosomal miR-451a correlates with SLE disease activity and renal damage. **(A)** The level of serum exosomal miR-451a in patients with LN was negatively correlated with systemic lupus erythematosus disease activity index score (*n* = 47, *r* = −0.616, *P* < 0.01). **(B)** The level of serum exosomal miR-451a in patients with LN was negatively correlated with 24-h urinary protein levels (*n* = 47, *r* = −0.649, *P* < 0.01). **(C)** The miR-451a level in exosomes from 47 patients with SLE with renal damage was significantly lower than that in 21 patients with SLE without renal damage by RT-qPCR. **(D)** Diagnostic utility of serum exosomal miR-451a to differentiate patients with SLE and renal damage from patients with SLE without renal damage. and the area under the ROC curve was 0.801 (95% confidence interval, 0.7–0.902; *P* < 0.001). **(E)** The expression level of miR-451a in renal proliferative type (*n* = 18) was lower than in non-proliferative type (*n* = 13), and the expression level of miR-451a in mix type (*n* = 16) was in the middle. **(F)** The expression level of exosomal miR-451a in SLE patients after treatment (*n* = 32) was significantly higher than that in those without treatment (*n* = 15). Treatment with traditional drugs, including glucocorticoid (*n* = 7) and hydroxychloroquine, increased the expression level of exosomal miR-451a. *P < 0.05, **P < 0.01, NS, no significance.

Recent studies have indicated that miR-451a was involved in the regulation of various human physiological and pathological processes (26). Furthermore, previous studies revealed that the levels of circulating miR-451a, which is important for erythropoiesis, were abnormal in the plasma of patients with SLE relative to normal controls, and miR-451a might be linked to abnormal erythropoiesis in SLE (9). Therefore, the present study evaluated the association between the levels of exosomal miR-451a in serum and the patient SLEDAI scores (Figure 4A) or 24-h urine protein quantity (Figure 4B). The level of serum exosomal miR-451a in patients with SLE was negatively linked to the SLEDAI score (*r* = −0.616, *P* < 0.01) and 24-h urine protein (*r* = −0.649, *P* < 0.01).

The clinical features of the study subjects were summarized according to renal dysfunction, and are presented in Table 1. Compared with patients with SLE devoid of renal damage, patients with SLE who have renal damage showed significantly downregulated serum exosomal miR-451a expression levels (*P* < 0.05) (Figure 4C). According to the degree of serum exosomal miR-451a expression in patients with SLE, an ROC curve was constructed to predict the manifestation of renal damage in patients with SLE, and the area under the ROC curve was 0.801 (95% confidence interval, 0.7–0.902; *P* < 0.001). When the miR-451a level was below 167.58, the sensitivity and specificity reached 70 and 100%, respectively, and these patients were considered to have renal damage (Figure 4D).

**Table 1:** Clinical data of the study subjects.

	**Renal damage**	**Without renal damage**	***P*-value**
**Demographic**			
Number, *n*	47	21	
Age, year, mean ± *SD*	29.0 ± 10.43542	34.2381 ± 10.00952	0.055
Female, *n* (%)	44 (94%)	21 (100%)	0.083
**Clinical and laboratory findings (mean** **±** ***SD*****)**			
SLEDAI	16.7200 ± 5.73937	8.1905 ± 2.13586	0.000
Serum WBC, 10^9^/L	6.6238 ± 4.04377	5.9300 ± 5.71946	0.563
Serum RBC, 10^12^/L	3.6086 ± 0.67606	3.9705 ± 0.57351	0.036
Hb, g/L	105.00 ± 21.68909	114.1429 ± 17.10307	0.091
PLT, 10^9^/L	183.00 ± 73.60197	167.3333 ± 82.63918	0.434
24-h proteinuria, mg	2377.3282 ± 2350.16894	39.5523 ± 15.59429	0.000
Serum C3, g/L	0.5576 ± 0.32405	0.5374 ± 0.23425	0.797
Serum C4, g/L	0.1169 ± 0.12103	0.1479 ± 0.11694	0.396
ALT, U/L	25.7837 ± 32.04501	51.9429 ± 129.95698	0.188
AST, U/L	32.2816 ± 49.15422	36.5353 ± 30.70588	0.740
TP	53.704 ± 13.06322	67.6650 ± 8.37347	0.000
ALB, g/L	26.968 ± 7.44595	38.3350 ± 6.86565	0.000
SCr, μ mol/L	125.854 ± 178.85081	48.8882 ± 10.77108	0.004
BUN, mmol/L	9.4376 ± 7.16294	3.7062 ± 0.90052	0.000
UA, μmol/L	371.8143 ± 105.26327	250.1846 ± 95.18854	0.000
dsDNA positive rate (%)	64	38.1	0.045

*ALB, albumin; ALT, glutamic-pyruvic transaminase; AST, glutamic oxalacetic transaminase; BUN, blood urea nitrogen; dsDNA, double-stranded DNA; Hb, hemoglobin; PLT, platelet count; RBC, red blood cell count; SCr, serum creatinine; SD, standard deviation; SLEDAI, systemic lupus erythematosus disease activity index; TP, total protein; UA, uric acid; WBC, white blood cell count*.

Based on renal pathology types of proliferative, non-proliferative and mixed, the expression levels of exosomal miR-451a were investigated, and it was found that the proliferative type showed the most significantly decrease (Figure 4E), which suggested that exosomal miR-451a acts as a biomarker in renal damage. To further understand the effects of various medications on the expression of exosomal miR-451a, the expression level of miR-451a in CD4^+^ T cells from patients with SLE treated with different drugs were analyzed, including seven patients treated with glucocorticoids, 18 patients treated with glucocorticoids combined with hydroxychloroquine, and 15 untreated patients. CD4^+^ T cells from 21 healthy controls were also included in this experiment. Notably, the expression level of miR-451a between normal controls and treated patients with SLE showed no statistical significance, and revealed a significant difference between normal controls, and the non-treated or treated and non-treated groups. Notably, glucocorticoid or hydroxychloroquine treatment elevated the expression level of exosomal miR-451a in CD4^+^ T cells (Figure 4F).

### Exosomal Shuttled miRNA-451a Is Involved in Intercellular Communication

**Figure 5:**
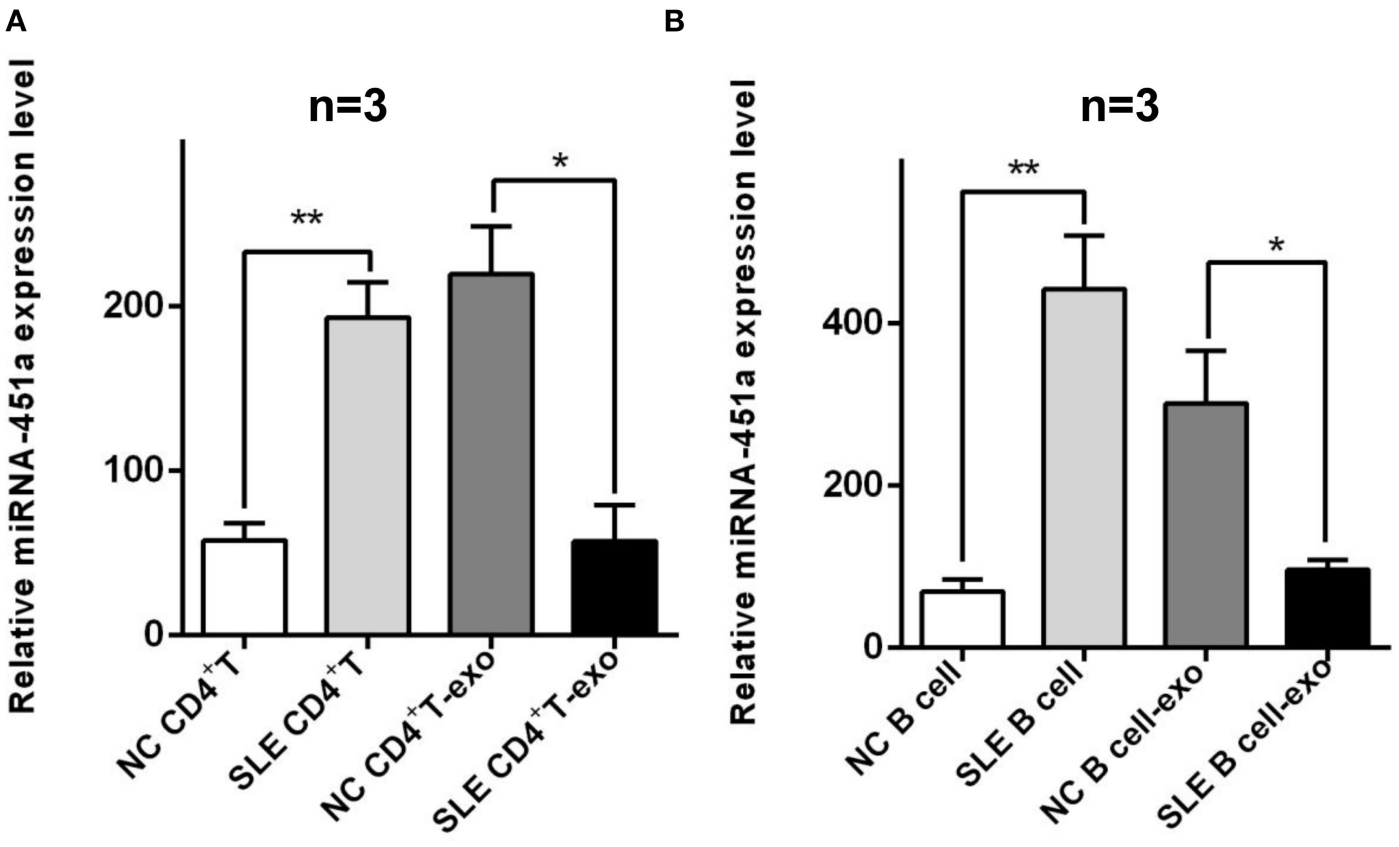
Differences verification of miR-451a expression in lymphocytes and lymphocytes derived exosomes. **(A)** The expression of miR-451a in CD4^+^ T cells of patients with SLE was significantly increased compared with that in normal CD4^+^ T cells, while the expression of T cells derived-exosomal miR-451a in patients with SLE was lower than that in normal controls (*n* = 3). **(B)** The expression of miR-451a in B cells or B cells derived-exosomes of SLE patients and normal controls showed the same trend (*n* = 3). *P < 0.05, **P < 0.01.

SLE is generally considered to be a crosstalk of abnormal differentiation of CD4^+^ T lymphocytes and abnormal secretion of cytokines, as well as abnormal activation of B cells and abnormal secretion of autoantibodies. Thus, studies on abnormal activation of T and B cells have become hot research topics. Based on the above results, we furtherly performed RT-qPCR to investigate the expression of miR-451a level in CD4^+^ T cells and T cells derived-exosomes of normal individuals and patients with SLE, and it was found that the expression of miR-451a in the CD4^+^ T cells of patients with SLE was significantly higher (*P* < 0.01) than that in normal individuals. In addition, the expression of miR-451a in SLE CD4^+^ T cell derived-exosomes (SLE CD4^+^ T cells-exo) was significantly reduced (*P* < 0.05) compared with that in normal CD4^+^ T cell-exo (Figure 5A). Similarly, the differences in miR-451a expression in B cells and B cells derived-exosomes of normal individuals and patients with SLE were compared, and the same results were observed (Figure 5B). This was consistent with our previous results of serum exosomal miRNA-451a. The maintenance of this expression difference in SLE patients may due to the selective mechanism of exosomes encapsulating miRNAs (27). In CD4^+^ T or B cells from patients with SLE, miR-451a preferentially enters exosomes, but the excretion of this kind of exosomes is reduced, resulting in high expression of miR-451a in cells and low expression of miR-451a in serum exosomes. The differential expression of miRNAs in serum exosomes and cells of SLE patients involves the cell communication between exosome-derived mother cells and T or B cells, inducing their abnormal immune response mechanisms, thereby participating in the occurrence and development of SLE.

**Figure 6:**
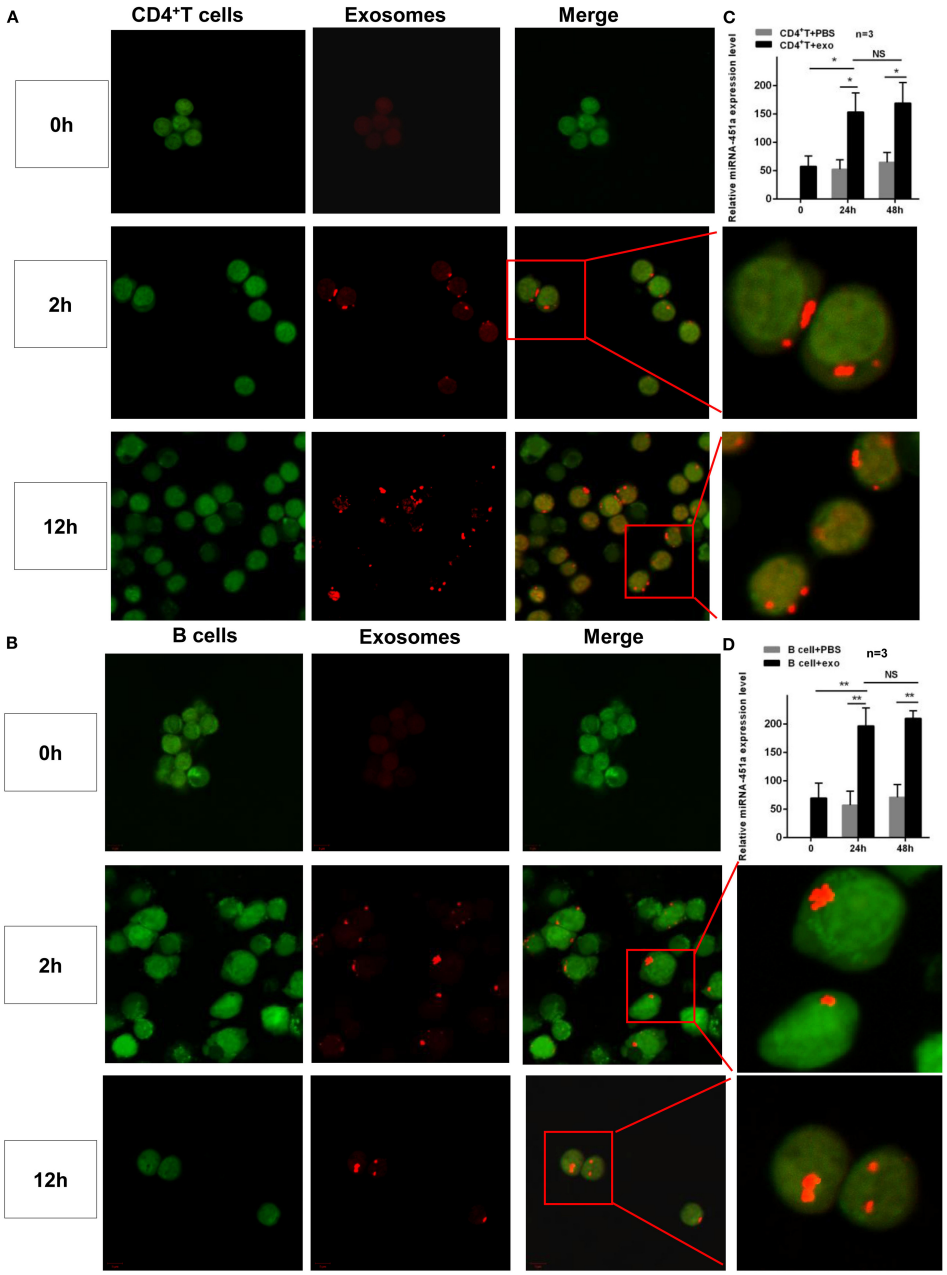
Dynamic observation of the effect of serum exosomes and target cells. Exo-Glow™-labeled exosomal RNAs were captured by **(A)** CD4^+^ T cells or **(B)** B cells entering the cytoplasm when cultured for 2 h *in vitro* and entering the nucleus when cultured for 12 h *in vitro*, as revealed by confocal laser microscopy. **(C)** Compared with that in CD4^+^ T cells before co-culture, the expression level of miR-451a in CD4^+^ T cells was significantly increased 24 h after co-culture of exosomes and CD4^+^ T cells, while it showed no difference at 24 and 48 h (*n* = 3). **(D)** The expression of miR-451a in B cells in an *in vitro* co-culture experiment showed the same trend (*n* = 3). *P < 0.05, **P < 0.01, NS, no significance.

Moreover, to investigate further the role of exosomes in SLE pathogenesis, the process of exosomes entering cells was directly observed by confocal microscopy. Normal CD4^+^ T and B cells were isolated and then co-cultured with normal serum exosomes or PBS, respectively. After 2 h, exosomes labeled with red fluorescence entering the cytoplasm(CD4^+^ T or B cells labeled with green fluorescence) were found, and after 12 h, exosomes labeled with red fluorescence entered the cell nucleus, while the control groups showed no fluorescent signal (Figures 6A,B). Simultaneously, in order to confirm whether miR-451a transported by exosomes played a role in cell communication, the changes in miR-451a expression in CD4^+^ T and B cells were compared before and after co-culturing of normal human serum exosomes with normal human CD4^+^T and B cells, respectively. It was found that expression level of miR-451a in CD4^+^ T cells increased significantly after co-cultivation for 24 h compared with that before co-culture (*P* < 0.05); there was no significant difference in the expression level of miR-451a between co-culture for 24 and 48 h (*P* > 0.05). The expression levels of miR-451a in the CD4^+^ T cell-exo group at 24 and 48 h were both significantly higher than those in the CD4^+^ T cell control group (both *P* < 0.05; Figure 6C). The same result was obtained in B cell co-culture (Figure 6D). This indirectly demonstrated that exosomes could transport miR-451a into CD4^+^ T and B cells, causing changes in miR-451a expression in cells, which might play a role in communication between lymphocytes.

## Discussion

Numerous techniques for purifying and detecting exosomal miRNAs exist (28–30). In this study, it was confirmed that the isolation system for exosomes and miRNAs generated a substance that was rich in serum exosomal miRNAs. Exosomes contain genetic material in the form of miRNAs, miRNAs and other non-coding RNAs that can easily move and function in other cells (18). Previous studies on exosomes have focused on exosomal miRNAs secreted by parental cells that have various biological functions, can target recipient cells and silence target mRNAs (27, 31, 32). The physical characteristics and targeting specificity of exosomal miRNAs are closely linked to the formation and progression of various diseases (13). These characteristics of circulating exosomal miRNAs have established their value as biomarkers in physiological and pathological conditions.

Although there are few reports on the association between urinary exosomal miRNA expression and LN, blood is a crucial medium that facilities the circulation of exosomes and transmission of cell signaling molecules to various parts of the body (19–22). Therefore, in the present study, a miRNA microarray profiling assay was conducted using serum from patients with SLE. The levels of exosomal miR-451a in serum were considerably reduced in patients with active SLE relative to those in patients with inactive SLE and control subjects. Furthermore, correlation analysis revealed that there was an inverse correlation between the level of exosomal miR-451a in the serum of patients with SLE and the SLEDAI score. Therefore, exosomal miR-451a in serum may be employed as a possible biomarker for assessing SLE activity.

LN has a considerable influence on the prognosis of SLE. Renal failure is a leading cause of SLE-related death (33). Our study next determined by RT-qPCR the levels of exosomal miR-451a in the serum of patients with SLE who exhibited renal damage and in a group of patients with SLE lacking renal damage. Our data demonstrated that, compared with patients with SLE exhibiting renal damage, patients with SLE without renal damage showed significantly downregulated serum exosomal miR-451a expression levels. Moreover, ROC curve analysis revealed that the serum exosomal miR-451a levels could distinguish with high sensitivity and specificity patients with SLE and renal damage from patients with SLE without renal damage, which suggests that exosomal miR-451a may serve as a novel potential biomarker for SLE renal damage.

Finally, correlation analysis also revealed that the serum level of exosomal miR-451a in patients with LN was negatively correlated with SLEDAI score and 24-h urinary protein levels. It was found that glucocorticoid or hydroxychloroquine treatment could increase the expression level of exosomal miR-451a in CD4^+^ T cells of patients with SLE. Therefore, it was concluded that exosomal miR-451a might serve as a treatment target in the future.

According to the classification criteria of the International Society of Nephrology/Renal Pathology for LN from 2003 (34), LN could be divided into types I-VI. Type III is focal LN; type IV is diffuse LN; and type V is membranous LN. The incidence of typed I, II and VI is low, and types I and II are usually mild clinically, and their treatment plan could be determined by the level of urine protein. Types III, IV, and V, as well as mixed type (types V+IV and V+III) are more common in clinical practice; their conditions are relatively severe; and their diagnosis and treatment guide is usually performed with kidney biopsy (35). Therefore, it is necessary to identify biomarkers with high sensitivity and specificity and low invasiveness to judge the pathological type and disease activity of LN in order to guide clinical treatment. Due to the different pathological types of LN perform different pathogenesis, there might be differences in the expression of cytokines in the peripheral blood of patients with LN (36–38). Therefore, in this study, 47 patients with active LN were divided into proliferative type (including types IV and III), non-proliferative type (type V) and mixed type (including types IV+V and V+III). By comparing the difference in expression of serum exosomal miR-451a, it was found that the proliferative type exhibited the most significantly decreased miR-451a expression level. Therefore, it was predicted that the expression of serum exosomal miR-451a is closely associated with disease activity, and is expected to become a biomarker for clinical judgment of LN pathological type and assessment of disease activity.

Cellular communication enables cells to receive signals from other cells and to function accurately, which is important for the development of multicellular organisms, immune adaptation and coordination of functions among different cell types within tissues (39, 40). Recent studies have shown that exosomes also played a critical role in signal transduction between cells (41–45). Exosomes can be secreted by a variety of cell lines and different cell types, including immune cells such as T cells, B cells, dendritic cells, and macrophages, as well as cardiovascular, tumor, stem and nerve cells (46, 47). Exosomes released by cells can communicate with neighboring or distant cells, delivering exosomal miRNAs to recipient cells (48). The function of exosomal miRNAs is generally considered to negatively regulate target genes to change their expression level (49–52). Previous studies have proposed that the pathogenesis of SLE is associated with abnormal activation of T cells that secrete cytokines, and with excessive secretion by B cells of autoantibodies, which participate in the initiation and maintenance of autoimmune diseases (53, 54). In this study, it was found that the difference in expression of exosomal miR-451a in CD4^+^ T or B cells and in serum of normal individuals and patients with SLE patients was markedly large, which might be associated with the selection mechanism of exosomes encapsulating miRNA (27). Emerging evidence indicated that miR-451a was involved in the regulation of various human physiological and pathological processes (26) and the level of circulating miR-451a was closely correlated with erythropoiesis formation, it was possible that miR-451a could be linked to the abnormal erythropoiesis of SLE (9). According to our current results, we used multiple international authoritative databases such as Mirbase/Targetscans/PicTar to predict the potential target gene of miRNA-451a, which hinted that MIF may act as a target gene. Published reports indicated that MIF was an autoimmune regulatory factor which was closely related to the pathogenesis of immune inflammatory diseases. MIF can further co-stimulate T and B cells, up-regulate the secretion of INF-γ, IL-6, TNF-α, etc. to form a positive feedback loop, maintain pro-inflammatory activity and inhibit the activation of apoptotic signaling pathways to be involved in the occurrence and development of SLE which may be our next research direction (55, 56).

## Conclusion

In summary, serum exosomes in active SLE patients could induce inflammatory immune response; miRNA microarray and verification RT-qPCR results validated the abnormal expression of exosomal miR-451a and miR-16 in serum from SLE patients, furtherly, downregulated serum exosomal miR-451a expression correlates with disease activity and renal damage. The serum exosomal miR-451a may serve as a potential biomarker for clinical determination of renal damage in SLE and different pathological types of lupus nephritis (LN). Moreover, conventional drug treatments including glucocorticoid and hydroxychloroquine could significantly increase the expression level of serum exosomal miR-451a. Finally, serum exosome miR-451a could be ingested by lymphocytes, and exosome miR-451a may play a role in the lymphocytes communication.

## Methods

### Subjects

The subjects enrolled in the present study (active SLE patients, *n* = 21; inactive SLE patients, *n* = 21; and healthy controls, *n* = 21) met the criteria of the American College of Rheumatology for SLE. The activity of the disease was assessed using systematic lupus activity measure (57) and systemic lupus erythematosus disease activity index (SLEDAI) (58) (active, score >5; inactive, score ≤4). Healthy control subjects were recruited volunteers, and were age- and sex-matched to the patients with lupus. In addition, 68 patients with SLE were recruited among dermatology and rheumatic immunology outpatients from our hospital (Table 1). Among them, 47 were patients with SLE and kidney damage. According to the SLEDAI-2000 scores, haematuria, albuminuria, tubular urine, and pyuria were at least one of the factors involved. That is, the urine sediment results of the patients collected 10 days before treatment was determined to have renal involvement if they met one or more of the following indexes: (i) Tubular urine: hemoglobin, granular, or erythrocyte tubular; (ii) haematuria excluding stone infection and other causes: red blood cell count >8,000 cells/ml; (iii) pyuria excluding infection: white blood cell (WBC) >5/HP; and (iv) new or recently increased proteinuria: >0.5 g/24 h. There were 21 patients with SLE without renal damage (according to the SLEDAI score, no haematuria, proteinuria, tubular urine, or pyuria; all four scores were 0). The current study was approved by the Human Ethics Committee of Third Xiangya Hospital, Central South University, and written informed consent was obtained from the participants.

### Exosome Isolation

Exosomes were extracted from serum using ExoQuick Exosome Precipitation Solution (System Biosciences) according to the protocol provided by the manufacturer. Serum was obtained by centrifugation (3,000 × g for 15 min) to remove cells and cell debris. In brief, 63 μl ExoQuick solution was mixed with 250 μl serum, followed by incubation at 4°C for 30 min. Exosome pellets were collected by centrifugation (1,500 × g for 30 min) at room temperature, and then dissolved in 50 μl nuclease-free water. Exosomes were extracted from T or B cells culture supernatant using ExoQuick-TC Exosome Precipitation Solution (System Biosciences). 2 ml ExoQuick-TC solution was mixed with 10 ml cell culture supernatant and the following experimental procedures were consistent with the serum exosome extraction method.

### Transmission Electron Microscopy (TEM)

Exosome pellets were resuspended in PBS and placed on a carbon-coated copper grid. After incubation for 5 min at room temperature, the exosomes were fixed with 2% paraformaldehyde, and then rinsed with water twice. The grid was negatively stained with 10% uranyl acetate for ~10 min. Next, the grid was observed and imaged by TEM (Tecnai G2 Spirit TWIN; FEI; Thermo Fisher Scientific, Inc.).

### Western Blotting

The isolated exosomes were washed once with PBS and then lysed in protein lysis buffer supplemented with a phosphatase inhibitor. Next, the lysates were centrifuged (14,000 × g for 15 min) at 4°C to collect the soluble protein. The isolated proteins were resolved on SDS-PAGE, and then subjected to western blotting according to standard procedures. The membranes were incubated with polyclonal anti-human TSG101 and CD63 antibodies (Bioworld Technology, Inc.) and then with goat anti-rabbit IgG conjugated to Sepharose beads (Bioworld Technology, Inc.). The bands were scanned and analyzed with Quantity One software (Bio-Rad Laboratories, Inc.).

### miRNA Extraction

The mirVana PARIS Kit (Ambion; Thermo Fisher Scientific, Inc.) was used to extract exosomal miRNA according to the protocol provided by the manufacturer. Exosome samples were placed on an enzyme-free worktable and incubated at room temperature (15–25°C) for 5–10 min. Added the 2X denaturing solution of the same volume and mixed it fully, added 1X PBS to 200 ul, then added 200 ul acid-phenol:chloroform every tube. After centrifugation (14,000 g, 30 min), the top layer is RNA. The subsequent operation was the same as the conventional RNA extraction process (22). NanoDrop 2000 (NanoDrop Technologies; Thermo Fisher Scientific, Inc.) was used to assess the quantity and quality of the extracted miRNA.

### miRNA Microarray Analysis

miRNA microarray profiles were obtained by comparing pools of patients with active SLE, patients with inactive SLE and healthy controls (each group of five samples mixed into one pool). The pooled samples (the minimum amount of RNA in each sample for Agilent miRNA microarray is not <0.5 ug) were sent to Shanghai Biotechnology Corporation (SBC) for Agilent miRNA Microarray 19.0 (Agilent Technologies, Inc.) hybridization and detection. Agilent 2100 Bioanalyzer was used for quality inspection and standard of quality control was For Agilent miRNA Chip & qPCR: RIN ≥ 6.0 and 28S/18S > 0.7; For others: RIN≥7.0 and 28S/18S > 0.7. Gene Spring Software 11.0(Agilent technologies, Santa Clara, CA, US) was used to normalize the data (Quantile Normalization). Statistical analyses were performed at SBC. Candidate miRNAs were selected based on their fold changes and potential research value in patients with SLE.

### miRNA Detection by Reverse Transcription-Quantitative PCR (RT-qPCR)

Exosomal miRNAs were reverse transcribed into cDNA using a reverse transcription kit (TaqMan MicroRNA; Applied Biosystems; Thermo Fisher Scientific, Inc.), and specific RT primers for each miRNA were obtained from the TaqMan microRNA assay kit (Applied Biosystems; Thermo Fisher Scientific, Inc.). RT-qPCR was performed using TaqMan Universal Master Mix II, no UNG (Applied Biosystems; Thermo Fisher Scientific, Inc.) and a LightCycler 96 Real-Time PCR System (Roche Diagnostics) as described previously. Differences in expression levels were determined using the 2^−ΔΔCq^ method. Cel-miR-39 (Takara Bio, Inc.) was used to normalize the technical variation between samples. All primer sequences showed in Table 2.

**Table 2:** Primer sequences for RT-qPCR.

**Names**	**Sequences**
Cel-miR-39	5′-AGCTGATTTCGTCTTGGTAATA-3′
miR-451a	F:5′-ACCGTTACCATTACTGAG-3′
	R:5′-GAACATGTCTGCGTATCTC-3′
miR-16	F:5′-AGCAGCACGTAAATATTGG-3′
	R:5′-GAACATGTCTGCGTATCTC-3′
IL-6	F:5′-AATTCGGTACATCCTCGACGGC-3′
	R:5′-GCCAGTGCCTCTTTGCTGCTTT-3′
TNF-α	F:5′-GGACACCATGAGCACTGAAAGC-3′
	R:5′-TGCCACGATCAGGAAGGAGAAG-3′
IFN-γ	F:5′-CATCCAAAAGAGTGTGGAGACA-3′
	R:5′-TGCTTTGCGTTGGACATTCAAG-3′
GAPDH	F:5′-GGAGCGAGATCCCTCCAAAAT-3′
	R:5′-GGCTGTTGTCATACTTCTCATGG-3′

### Total CD4^+^T Cells or B Cells Isolation

PBMCs were separated from the peripheral blood of healthy controls and SLE patients by density gradient centrifugation (GE Healthcare, Switzerland). CD4^+^T cells(CD4^+^T cell positive sorting kit) or B cells (CD19 cell positive sorting kit) were isolated by positive selection using Miltenyi beads(Germany) (80 ul buffer solution and 20 ul MACS CD4 magnetic beads were added for every 10^7^ cells) according to the manufacturer's instructions (Miltenyi, Germany) in sterile or non-sterile conditions. Then, the cells were cultured or collected for subsequent experiments.

### Co-culture

Serum exosomes were extracted from healthy subjects or SLE patients and suspensed with PBS, then added to the 24-well plate according to the ratio of 10 ul/5^*^10^5^ cells every well. The samples were cultured in an incubator at 37°C and 5% CO_2_ for 24 h and cell precipitation was collected for subsequent experiments. In confocal assay, the experimental group was treated with normal serum exosomes co-cultured with CD4^+^T cells of normal subjects and the control group was treated with equal sterile PBS co-cultured with CD4^+^T cells of normal subjects. The re-suspended exosomes are added to 6-well plates at different time points with a minimum of 100 ul/ well. Exosomes and CD4^+^T were co-incubated for 2–24 h, and then observed under a laser confocal microscope.

### Flow Cytometry

Cytokines and surface markers were assessed by flow cytometry with a FACS Canto II (BD Biosciences). After sorting CD4^+^T cells, First, we used phorbol-12-myristate-13-acetate (PMA) and ionomycin with the addition of GolgiPlug (BD Biosciences) to stimulate and activate the cells for 4 h for cytokines detection, including IFN-γ, IL-6, and TNF-α. Surface marker staining was done at 4°C in the dark for 30 min. The following antibodies were used for flow cytometry: anti-human CD4-FITC (clone: RPA-T4), IFN-γ-PercpCy5.5 (clone: B27), IL-6-PE (clone: MQ2-13A5), and TNF-α-PE (clone: MAb11) were purchased from BD Biosciences. Events were collected and analyzed with FlowJo software (Tree Star, Inc.).

### Confocal Microscopy

CD4^+^ T cells and B cells were sorted and stained with CFSE (BD Biosciences), while the Exo-Glow kit (SBI, USA) fluorescently labeled RNAs in exosomes. CD4^+^ T cells were washed repeatedly with PBS and added CFSE reagent (configured to final concentration of 10 μM) to bath at 37°C for 10–15 min. As for exosomal RNAs staining, exosomes were re-suspended to 500 ul in volume with PBS, and 50 ul Exo-Red (Exo-Glow kit) was added to the re-suspended exosomes, after mixed, it was bathed at 37°C for 10 min. Then added 100 ul Exo-TC to stop the reaction. Events were collected and analyzed with FlowJo software (Tree Star, Inc.). CFSE-labeled CD4^+^ T cells appeared as green light, while exo-red marked exosomal RNAs with red light.

### Statistical Analyses

All of the diagrams and graphs analysis was conducted using GraphPad Prism 6.0 (GraphPad Software, Inc.) and the data are presented as the average value ± SEM. We assessed data for normal distribution and similar variance between groups. Student's *t*-test was used to compare the mean of two independent samples with normal distributions. The Mann-Whitney *U*-test was conducted to compare the variables that were not normally distributed. Correlation and partial correlation analyses were performed by using Pearson's *r*-test or Spearman's *r*-test. A receiver operating characteristic (ROC) curve was constructed to evaluate the occurrence of renal damage in patients with SLE. *P* < 0.05 was considered to indicate a statistically significant difference.

## Standard Biosecurity and Institutional Safety Procedures

All the biosafety measurements have been adopted and the institutional safety procedures are adhered. The laboratory of our institution has biosafety level 1 (BSL-1) standard where all standards and protocols are adopted as per the guidelines of CLSI.

## Data Availability Statement

The raw data supporting the conclusions of this article will be made available by the authors, without undue reservation.

## Ethics Statement

The studies involving human participants were reviewed and approved by the Human Ethics Committee of Third Xiangya Hospital, Central South University. The patients/participants provided their written informed consent to participate in this study.

## Author Contributions

JZ conceived and designed the study. LT performed the experiments and wrote the paper. YZ and LG analyzed the data. QL, MZ, HW, XT, and LZ reviewed and edited the manuscript. All authors read and approved the manuscript.

## Conflict of Interest

The authors declare that the research was conducted in the absence of any commercial or financial relationships that could be construed as a potential conflict of interest.

## Abbreviations

LN: lupus nephritis
ROC: receiver operating characteristic
RT-qPCR: reverse transcription-quantitative PCR
SBC: Shanghai Biotechnology Corporation
SLE: systemic lupus erythematosus
SLEDAI: systemic lupus erythematosus disease activity index
TEM: transmission electron microscopy.
